# Synergistic effect of LCI with ESWT on treating patients with mild to moderate CTS: a randomized controlled trial

**DOI:** 10.1186/s13018-023-03940-0

**Published:** 2023-07-01

**Authors:** Morteza Gholipour, Sona Bonakdar, Mona Gorji, Reza Minaei

**Affiliations:** 1 Clinical Research Development Unit of Akhtar Hospital, Shahid Beheshti University of Medical Sciences, Tehran, Iran; 2 Skin Research Center, Shahid Beheshti University of Medical Sciences, Tehran, Iran

**Keywords:** Carpal tunnel syndrome, Extracorporeal shock wave therapy, Injection, Pain

## Abstract

**Background:**

Applying radial extracorporeal shock wave therapy (R-ESWT) with LCI(local corticosteroid injection) in carpal tunnel syndrome (CTS) management is gaining momentum. The objective is to actualize the topic of this study.

**Methods:**

In this prospective randomized controlled trial, forty patients with mild to moderate CTS are divided into two sham- R-ESWT and R-ESWT groups subject to LCI(local corticosteroid injection). The first group received four sessions of sham-ESWT weekly, which involved sound but no energy; the second group received R-ESWT at equal intervals and were assessed for pain score (VAS score) and symptoms (GSS) baseline, 1st month, 3rd month, and 6th month.

**Results:**

A considerable improvement is observed in both groups for pain at (*P* < 0.05) and symptoms at (*P* < 0.05) in the 3rd month. The second group revealed more significant symptom improvement at (*P* < 0.05) in the 6th month.

**Conclusion:**

The R-ESWT + LCI combined therapy course is the first line of treatment in patients with mild to moderate symptoms and leads to control and reduction of symptoms and the need for surgery, thus a primary concern in CTS treatment with an orthopedist.

## Introduction

Carpal tunnel syndrome (CTS), which appears by the pressure exerted on the median nerve in the carpal tunnel, is the most common peripheral neuropathy. Edema, tendonitis, and hormonal changes like hypothyroidism and menopause are involved and manual activity can play a role in increasing nerve compression [1–4]. The syndrome occurs with symptoms like paresthesia, dysesthesia, anesthesia, weakness, and atrophy of the thenar muscle. These symptoms are usually concentrated in hand but can extend to the forearm, arm, and even shoulder [5, 6]. The risk factors for CTS are diabetes, menopause, hypothyroidism, obesity, osteoarthritis, pregnancy, and smoking [7]. The diagnosis of CTS is mainly based on history, clinical findings, and examination through the Tinel's sign and Phalen test [8]. The nerve conduction velocity (NCV) electrodiagnostic tests are usually run to confirm the diagnosis or differentiation from other diseases.

Electrohydraulic shockwaves are the high-energy acoustic waves generated by the underwater explosion with high voltage electrodes. These shockwaves are of two types: (1) Radial shockwaves, the surface shocks, which are better for bigger treatment areas of superficial indications. Radial shockwaves are emitted through a pneumatic mechanism. Compressed air fires a projectile, which in turn strikes a metal tool called a transmitter. This impact emits a wave that is transmitted radially, dissipating its intensity as it goes through the different tissue layers. (2) Focused shockwaves, the hard shocks penetrate deeper into the tissues than Radial shockwaves and target one specific area. The Focused shockwaves are beneficial to tissues close to the bone calcifications and non-unions. This shockwave is defined as a noninvasive procedure with a sequence of single-wave pulses at (100 MPa) pressure and (G10 Nsecs) velocity over a short period of (10 Kiloseconds) time generated on the body and concentrated in a specific part of the body [9, 10]. Many studies have revealed that this modality/shockwave is an effective and lasting way to reduce pain in soft tissue diseases like plantar fasciitis and Achilles tendinopathy[11]. The inflammation in soft tissues is reduced through biochemical changes like nitric oxide (NO)[12, 13]. ESWT rapidly increases endothelial NO synthase (eNOS) activity in the treated cells. The first line of treatment approach in patients with mild to moderate CTS is patient education [14]. Changes in habits like restricting wrist movement and reduced activity Heavy workloads should be considered as the first-line approach. Many conservative treatments exist, like wrist splints, steroid injections, and laser treatments, with limited effectiveness [15, 16]. The ESWT is a practical short-term noninvasive treatment for mild to moderate CTS and improves it. Our theory is that ESWT in combination with LCI is effective for reducing pain and improving symptoms in mild to moderate patients.

## Methods and materials

This is a prospective clinical trial run from February to August 2020 on 47 patient within the 30 to 60 age range with paresthesia, dysesthesia, and thenar muscle weakness, in Akhtar Hospital (Tehran), who tested positive for Phalen and Tinel test. The tests’ outcomes are confirmed by neurophysiological tests (EMG-NCV) for mild to moderate CTS.

Severity of CTS is as follows: normal (grade 0); very mild (grade 1), CTS demonstrable only with most sensitive tests; mild (grade 2), sensory nerve conduction velocity slow on finger/wrist measurement, normal terminal motor latency; moderate (grade 3), sensory potential preserved with motor slowing, distal motor latency to abductor pollicis brevis (APB) < 6.5 ms; severe (grade 4), sensory potentials absent but motor response preserved, distal motor latency to APB < 6. 5 ms; very severe (grade 5), terminal latency to APB > 6.5 ms; extremely severe (grade 6), sensory and motor potentials effectively unrecordable (surface motor potential from APB < 0.2 mV amplitude)[17].

The exclusion criteria consist of diagnosis of sensory and/or motor neuropathy other than CTS, previous wrist trauma, surgery for CTS, treatment with ultrasound, ESWT, or local corticosteroid injection, pregnancy, infection at the treatment site, scar burn, and systemic diseases (Rheumatoid arthritis-lupus erythema-scleroderma).

The study protocol is subject to the Institutional Review Board and the Ethics Committee of Shahid Beheshti University of Medical Sciences regulations, which are explained to the participants[IR.SBMU.RETECH.REC.1399.1150]. Applying night splints and other oral medications is prohibited during the course, and all patients sign an informed consent. The subjects are labeled and randomly assigned through a random assignment sequence generated by the software to group 1 (sham -ESWT) and group 2(ESWT).

## Treatment protocol

The triamcinolone acetone (1 ml) + lidocaine (1 ml) is injected into all the patients between the palmaris longus tendon (PL) and the flexor carpi ulnaris tendon (FCU) in the wrist area once [18]. Local corticosteroid injection was performed 24 h later to prevent sensitivity and complications at the ESWT site. Each patient is subjected to the ESWT (electromagnetic standard DUOLITH SD1, Storz Medical, Tägerwilen, Switzerland) device. In the second group, ESWT is performed in the first session at 2600 beats average (with focusing probe) and a very low, 0.03 mj / mm2 energy flux density. Depending on patient tolerance, this energy follows a gradual incremental pattern for the next three sessions. The pulse repetition frequency is 4 Hz. In the (sham-ESWT) group, the ESWT device waves less and generates sound. At this stage, the patient is seated with the arm on the table and the palm facing up, and the ESWT probe is held vertically to the zone between the thenar and hypothenar ridges.( first session at 2600 beats average (with focusing probe) and a very low, 0.03 mj / mm2 energy flux density).

All participants underwent clinical follow-up before beginning the treatment, at the end of the 1st, 3rd, and 6th months for VAS scores, and filled out the GSS questionnaire. This questionnaire covers the pain, numbness, paresthesia, weakness/clumsiness, and nocturnal waking [19]. The scale of GSS begins from 0 (no symptoms) to 10 (very severe), with the 50 as the worst score. The pain severity is measured through the Visual Analog Scale (VAS) [20], where 0 and 10 indicate no pain and the most severe imaginable pain, respectively. All treatments are run by a team of one orthopedic and one physiotherapist. At the end of the 6th month, patients with exacerbation of paresthesia, finger tingling, and decreased strength symptoms are referred for surgery after being confirmed by the EMG-NCV. For patients who were treated with placebo, in case of a significant lesion or the need for early surgery, all treatment costs will be paid for the patients.

### Statistical analysis

The statistical analyses are run in SPSS software (SPSS, Inc., Chicago, IL, USA, Version 16) with a significance level of 5% and a 95% confidence interval. Descriptive data are reported as the mean ± SD. A chi-square test is run for qualitative variables, and a student’s t test is run to compare pain and Global symptom scores between the subject groups. Repeated measurements of ANOVA are applied to compare the Visual Analog Scale score and Global Symptom score trends within and between the groups.

## Results

**Fig. 1:**
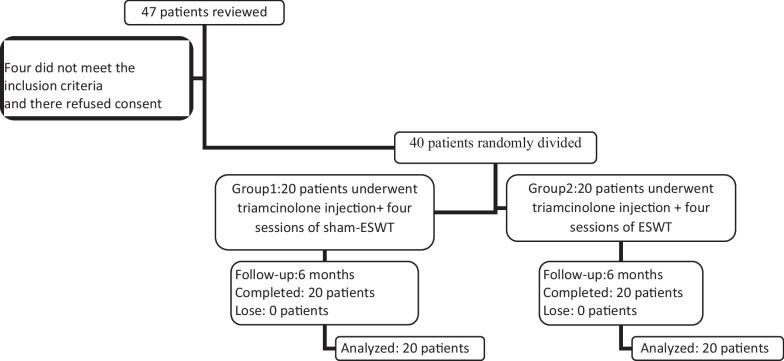
The study flowchart

Forty-seven patients are considered eligible for the study. After the inclusion and exclusion criteria, 40 patients are selected and randomized into: (Sham-ESWT) (20patients, 20 wrists) and (ESWT) (20 patients, 20wrists) groups, Fig. 1. No adverse events are recorded during the study period, and all patients completed the six months of follow-up and underwent the final analysis, Fig. 1. The Sham-ESWT group consists of 15 females (75%), with 44.90 ± 10.42 age average, and the ESWT group, with 18 females (90%) with 45.15 ± 9.22 age average. The groups were similar in age, gender, the proportion of dominant hand lesions, and duration of symptoms (*P* > 0.05), Table 1.

**Table1:** Patients’ Demographic

Variable	Sham-ESWT Group	ESWT Group	*P* Value
Age(years)	44.90 ± 10.42	45.15 ± 9.22	0.423
Gender			0.885
Male	5 (25%)	2 (10%)	
Female	15 (75%)	18 (90%)	
Dominant hand lesion%	80%	70%	0.465
Duration of symptoms(weeks)	14.80 ± 2.83	14.95 ± 2.43	0.290

^*^P values of 0.05 or less are considered statistically significant

**Fig. 2:**
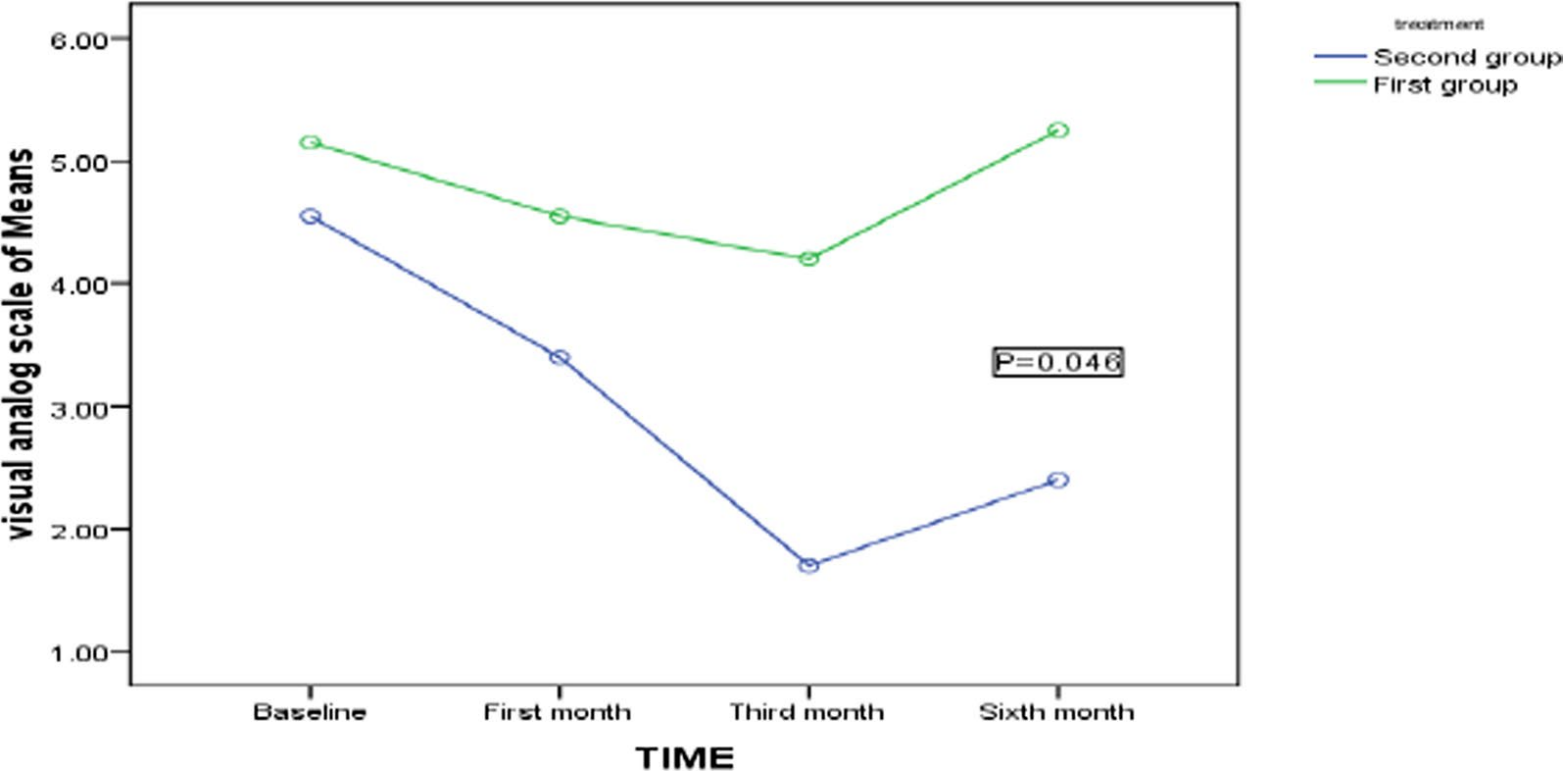
Trend of pain VAS score during the study period between two groups by repeated measurements of ANOVA. *The first group is Sham-ESWT, and the second group is ESWT

**Fig. 3:**
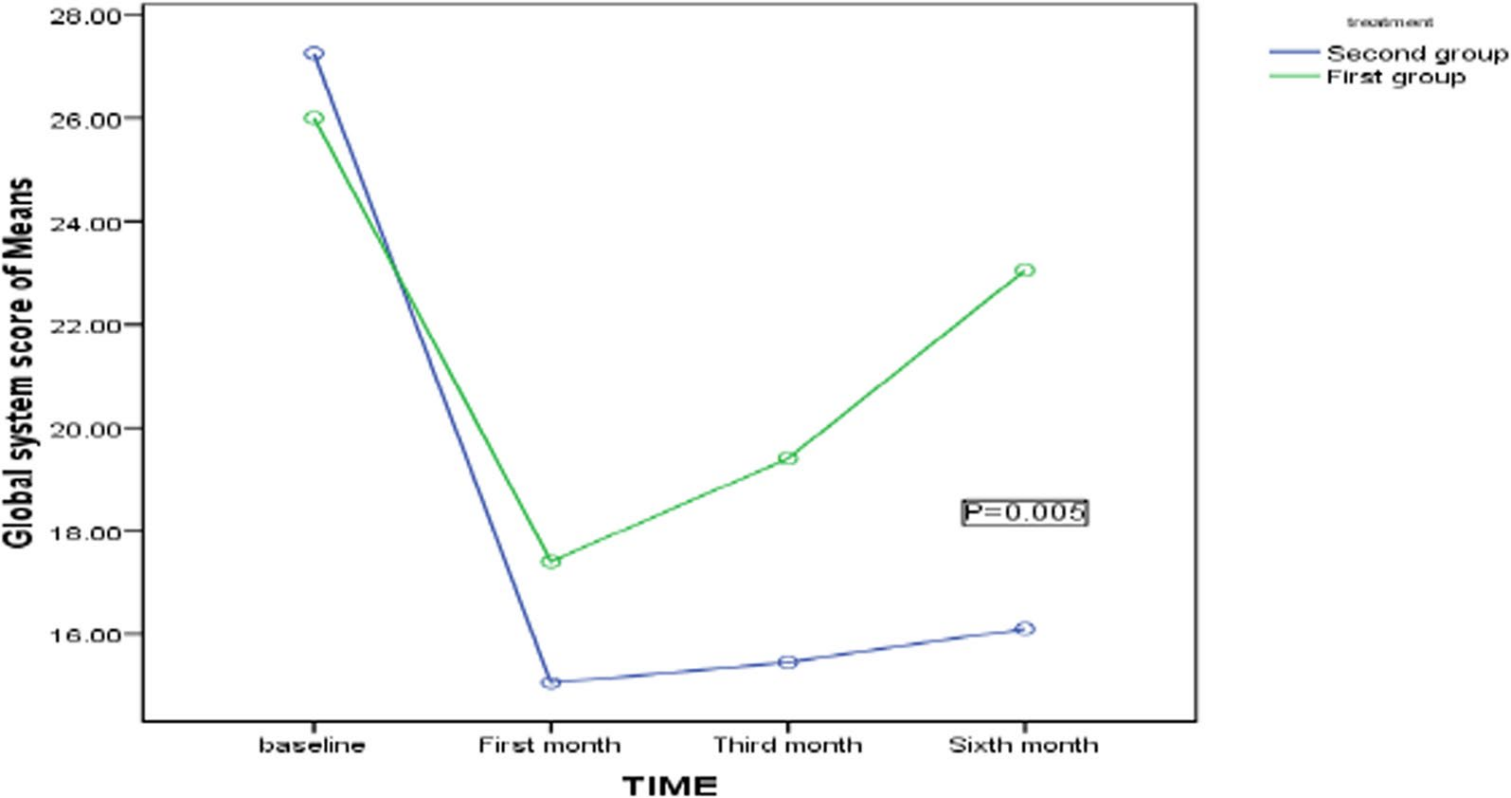
Trend of Global System score during the study period between two groups by repeated measurements of ANOVA. *The first group is Sham-ESWT, and the second group is ESWT

The groups' pain VAS and GSS scores at the beginning, 1st, 3rd, and 6th months are compared. The pain VAS score is similar in both groups at the beginning, but after the 1st month, no statistically significant difference is observed between the groups at (*p* = 0.677). In the 3rd month, the pain score in the ESWT group is recorded as significantly lower than the sham-ESWT group at (*p* = 0.006). At the end of the follow-up, this score reveals a statistically significant difference between the groups at (*P* = 0.008). GSS score is not significantly different between groups at the 1st follow-up at (*P* = 0.486); after one month, the same holds at (*P* = 0.401).In the 3rd month, this score in the ESWT group is (15.45 ± 1.53) and in the sham-ESWT group is (19.40 ± 3.11) at (*P* = 0.002). At the end of the follow-up, this score reveals a statistically significant difference between the two groups at (*P* = 0.007), Table 2. Pain score in both groups decreases significantly during the study period, more in the ESWT group at (*P* = 0.046) than in the sham-ESWT group, Fig. 2, though there exists a difference in their trend at (*P* = 0.005), Fig. 3. This decrease in both groups during the study period has statistical significance. At the end of the study period, 15 (75%) patients from the sham-ESWT group and 8 (40%) from the ESWT group are referred for carpal tunnel release, at (*P* = 0.025) statistically significant where fewer patients in the ESWT group require surgery.

**Table 2:** The VAS and GSS of the subject groups compared

Variable	Sham-ESWT Group	ESWL Group	*P* Value
GSS score baseline	26.00 ± 4.99	27.25 ± 4.76	0.486
GSS score (1st month)	17.40 ± 3.28	15.05 ± 2.35	0.401
GSS score (3rd month)	19.40 ± 3.11	15.45 ± 1.53	**0.002**
GSS score (6th month)	23.05 ± 3.51	16.10 ± 1.77	**0.007**
VAS score baseline	5.15 ± 1.46	4.55 ± 1.35	0.644
VAS score(1st month)	4.55 ± 1.14	3.40 ± 1.09	0.677
VAS score(3rd month)	4.20 ± 1.50	1.70 ± 0.80	**0.006**
VAS score(6th month)	5.25 ± 1.83	2.4 ± 0.99	**0.008**
Surgery required	15(75%)	8(40%)	**0.025**

^*^P values of 0.05 or less are considered statistically significant

## Discussion

ESWT is a new noninvasive procedure applied extensively in recent years in treating soft tissue diseases like osteoarthritis [21] and peripheral neuropathy [16, 22, 23]. Based on the findings here, patients in both groups had almost similar result in the early stages of follow-up concerning VAS and GSS criteria, while in the final stages, the second group showed better results. In another study [24, 32] the comparison with night splint plus local corticosteroid injection and ESWT showed a better symptom relief at 12 weeks with local corticosteroids. Although different performance criteria are applied in this study, the results correspond to that of the available studies. There is a lack in the literature of follow-up after ESWT treatments over the 6-month time [25, 25]. In a recent systematic review and meta-analysis on ESWT [26, 26] in carpal tunnel syndrome, on 376 patients, ESWT did not show superior efficacy compared to treatment with night wrist splint alone at 2 and 3 months follow-up. The effect of LCI in treating mild to moderate carpal tunnel syndrome is evident. Meys et al. [27] assessed 113 patients with carpal tunnel syndrome with less severe swelling on ultrasound, and the effect of single-dose corticosteroid injections revealed that after 67 follow-up periods of 12 months, about 67. 4% of patients required surgery. ESWT was first applied in the treatment of carpal tunnel syndrome [22], where it revealed that, according to the Levin-Boston questionnaire, statistically, the effect of one ESWT session was equal to that of one LCI session in CTS treatment at (*P* < 0.05). The results of this study revealed that, in the short term, approximately five months after the end of treatment, patients with mild to moderate CTS, not surgery candidates, may benefit from the synergy of ESWT with LCI. Atthakomol et al. [28] reported that patients treated with ESWT have significantly lower VAS and the Boston Carpal Tunnel Questionnaire scores compared with the LCI group in 12 and 24 weeks of follow-up. Celik et al. [29] revealed that patients significantly improved VAS scores in the 1st month, while the same increased statistically in the 3rd and 6th months. Milo et al. [30] exhibited that in 14 patients with carpal tunnel syndrome treated with injectable corticosteroids, the clinical results are satisfactory, and the VAS score decreased significantly after one month at (*P* < 0.05) but increased over 6th month, still less than the initial value, which corresponds with this study. Similar findings are evident in [28, 31]. Due to the temporary anti-inflammatory effect of injectable steroids without changing the underlying cause of the disease, injection therapy lacks long-term efficacy.

In many studies, nitric oxide produced by ESWT contributes to an increase in the angiogenesis growth factors’ level and inhibits inflammation through the suppressive production of pro-inflammatory cytokines [12, 13, 32, 33]. Takahashi et al. reported [34], the second and subsequent sessions have a cumulative effect on a neuronal filament with longer analgesic effects. That the patients with mild to moderate carpal tunnel syndrome with pain and disability could merely benefit from 3 sessions of ESWT for at least 3 months, compared with ultrasound and cryopreservation is revealed by [15]. That the effect of 3 sessions of combined ESWT with nocturnal splint or isometric tendon training in patients with CTS for at least six months compared with a diet consisting of (Echinacea angomedolia, alpha lipoid acid, linoleic acid, and quercetin) significantly improves pain, the severity of symptoms and functional scores, and electrodiagnostic results are revealed in [29]. They concluded that shock in association with ALA, GLA, and Echinacea due to its antioxidant effect is an effective treatment to control symptoms and improve the development of CTS. Wu et al. [35] in first assessed the ESWT in a prospective, randomized, double-blind, placebo-controlled study and found that the benefit of ESWT in treating CTS becomes apparent after the 3rd follow-up.

It can be deduced that this study is the first where corticosteroids are consumed as a supplementary with ESWT in patients with mild to moderate CTS; consequently, this study is subject to many limitations, like a small statistical population and short-term follow-up period. Another important restricting component here is the gender with a high count of females; if the same were males, the results might have varied. Accordingly, evaluation of the components that would indicate the possible mechanisms of ESWT and corticosteroids’ simultaneous action in future studies are of primary concern.

## Conclusions

The findings of this study showed that patients with mild to moderate carpal tunnel syndrome treated with R-ESWT are less candidates for surgery than patients with only LC injection. Because R-ESWT is noninvasive, it is ideal for treating and controlling symptoms. R-ESWT is recommended as a noninvasive first-line treatment, while surgery may be required in cases of recurrence.

## Data Availability

Not applicable.
